# In vivo insertion pool sequencing identifies virulence factors in a complex fungal–host interaction

**DOI:** 10.1371/journal.pbio.2005129

**Published:** 2018-04-23

**Authors:** Simon Uhse, Florian G. Pflug, Alexandra Stirnberg, Klaus Ehrlinger, Arndt von Haeseler, Armin Djamei

**Affiliations:** 1 Gregor Mendel Institute (GMI), Austrian Academy of Sciences, Vienna BioCenter (VBC), Vienna, Austria; 2 Center for Integrative Bioinformatics Vienna (CIBIV), Max F Perutz Laboratories (MFPL), University of Vienna, Medical University Vienna, Vienna, Austria; 3 Bioinformatics and Computational Biology, Faculty of Computer Science, University of Vienna, Vienna, Austria; Duke University Medical Center, United States of America

## Abstract

Large-scale insertional mutagenesis screens can be powerful genome-wide tools if they are streamlined with efficient downstream analysis, which is a serious bottleneck in complex biological systems. A major impediment to the success of next-generation sequencing (NGS)-based screens for virulence factors is that the genetic material of pathogens is often underrepresented within the eukaryotic host, making detection extremely challenging. We therefore established insertion Pool-Sequencing (iPool-Seq) on maize infected with the biotrophic fungus *U*. *maydis*. iPool-Seq features tagmentation, unique molecular barcodes, and affinity purification of pathogen insertion mutant DNA from in vivo-infected tissues. In a proof of concept using iPool-Seq, we identified 28 virulence factors, including 23 that were previously uncharacterized, from an initial pool of 195 candidate effector mutants. Because of its sensitivity and quantitative nature, iPool-Seq can be applied to any insertional mutagenesis library and is especially suitable for genetically complex setups like pooled infections of eukaryotic hosts.

## Author summary

Insertion mutant screens are widely used to identify genotype–phenotype relationships. In negative selection screens, a major limitation is the efficient identification of mutants that are lost or retained after selection. To identify these mutants, the two genomic sequences flanking the insertion cassette must be found. However, pinpointing these insertion flanks within a genome is like looking for a needle in a haystack; a problem that becomes even worse when several organisms form a biotrophic interaction. To overcome this hurdle, we developed insertion Pool-Sequencing (iPool-Seq). With iPool-Seq, we were able to efficiently amplify and enrich insertion flanks from complex genomic DNA samples. This technique allows for the quantification of relative insertion mutant abundance before and after selection by next-generation sequencing (NGS). We demonstrate the power of iPool-Seq with a negative selection screen by infecting maize with 195 candidate effector mutants of the fungal pathogen *Ustilago maydis*. We identified 28 virulence factors, of which 23 have not been previously described. iPool-Seq is extremely sensitive, cost- and time-efficient, and promises to be a powerful tool for identifying target genes in large selection screens.

## Introduction

Virulence factors are key for successful infections by pathogens. Their identification is of major interest because of the necessity to develop effective counter strategies. For instance, fungal virulence factors are typically identified by mutating single loci in fungi, followed by individual fungal mutant infections of host tissue and subsequent assessment of pathogen fitness [1–4]. Individual infection assays are not ideal for the genetic screening of a large number of pathogen mutants because they are laborious, cost-intensive, and—most importantly—assessment of infections is often subjective and qualitative rather than quantitative. An attractive alternative is infection with a pool of pathogen mutants allowing direct assessment of individual pathogen fitness in the same host tissue. However, using a pooled pathogen infection creates the challenge of identifying pathogens with reduced virulence within a complex mixture of genetic material extracted from infected host tissue.

Mutant collections can be efficiently generated using insertional mutagenesis. Insertional mutagenesis employs gene cassettes that commonly comprise a selectable marker under the control of a strong constitutive promoter. The detection of genome–cassette junctions can serve as a molecular identifier for each insertion mutant. During screening, insertional mutants before selection in the host are defined as the genetic input, whereas surviving insertional mutants after selection comprise the genetic output. Insertional mutagenesis can be achieved randomly through transposon insertion [5–8] or *Agrobacterium tumefaciens*-mediated transformation [3, 9], or specifically through site-specific insertion by homologous recombination [10, 11].

Over the last decade, several approaches were established that use massive parallel sequencing for the detection of inserted gene cassettes. These approaches were successfully used to track mutants from the small genomes of prokaryotic pathogens and allowed the identification of bacterial genes involved in virulence or host colonization after pooled infections [12–16]. However, only a few attempts were reported that identified virulence factors using pools of eukaryotic pathogens [17]. The main factors limiting the successful insertional mutagenesis of eukaryotic pathogens by pooled infections in complex host-pathogen systems are variable infection rates of individually mutated pathogens, the size ratio of host/pathogen genomes, the inability to sufficiently detect inserted gene cassettes from pathogenic material, and biases that arise through PCR-based amplification steps.

To enable successful and quantitative insertion mutant screen-based identification of virulence factors in complex biological systems, we developed insertion Pool-Sequencing (iPool-Seq). We determined the sensitivity and efficiency of iPool-Seq using an insertion mutant collection of 195 predicted virulence factors encoded by the maize pathogen *U*. *maydis*. The haploid *U*. *maydis* genome consists of approximately 20.5 megabases [18, 19], whereas the diploid genome of maize is 2.3 gigabases large [20]. This represents a 100-fold genome size difference, which is beside the proportion between fungal and host plant genome abundance as a limiting factor, making the robust detection of *U*. *maydis* sequence information in infected maize tissue necessary. The iPool-Seq workflow consists of Tn5 Transposase-mediated tagmentation of complex genomic DNA (gDNA) allowing efficient library preparation from low-input material [21, 22]. This is followed by the efficient enrichment of extremely rare insertion cassettes from fungal genomes using biotin-streptavidin affinity purification of PCR products. Amplification biases are monitored through incorporated unique molecular identifiers (UMIs). Insertional mutant fitness within host tissues is directly measured through quantification of UMI counts present in infected output material compared to UMI counts from the input library.

iPool-Seq on *U*. *maydis* infections of maize confirmed the identity of 5 known fungal virulence factors that were included as positive controls in the screen. Importantly, 23 previously unreported virulence factors encoded by *U*. *maydis* were uncovered. Three of these factors were confirmed to be novel virulence factors of *U*. *maydis* after testing by individual infection. The combination of pooled insertion mutant infections and iPool-Seq technology represents a straightforward and cost-effective approach to map insertion mutants in complex host–pathogen systems with the potential to generate genome-wide virulence maps of relevant crop pathogens and beyond.

## Results

### iPool-sequencing design and library generation

**Fig 1:**
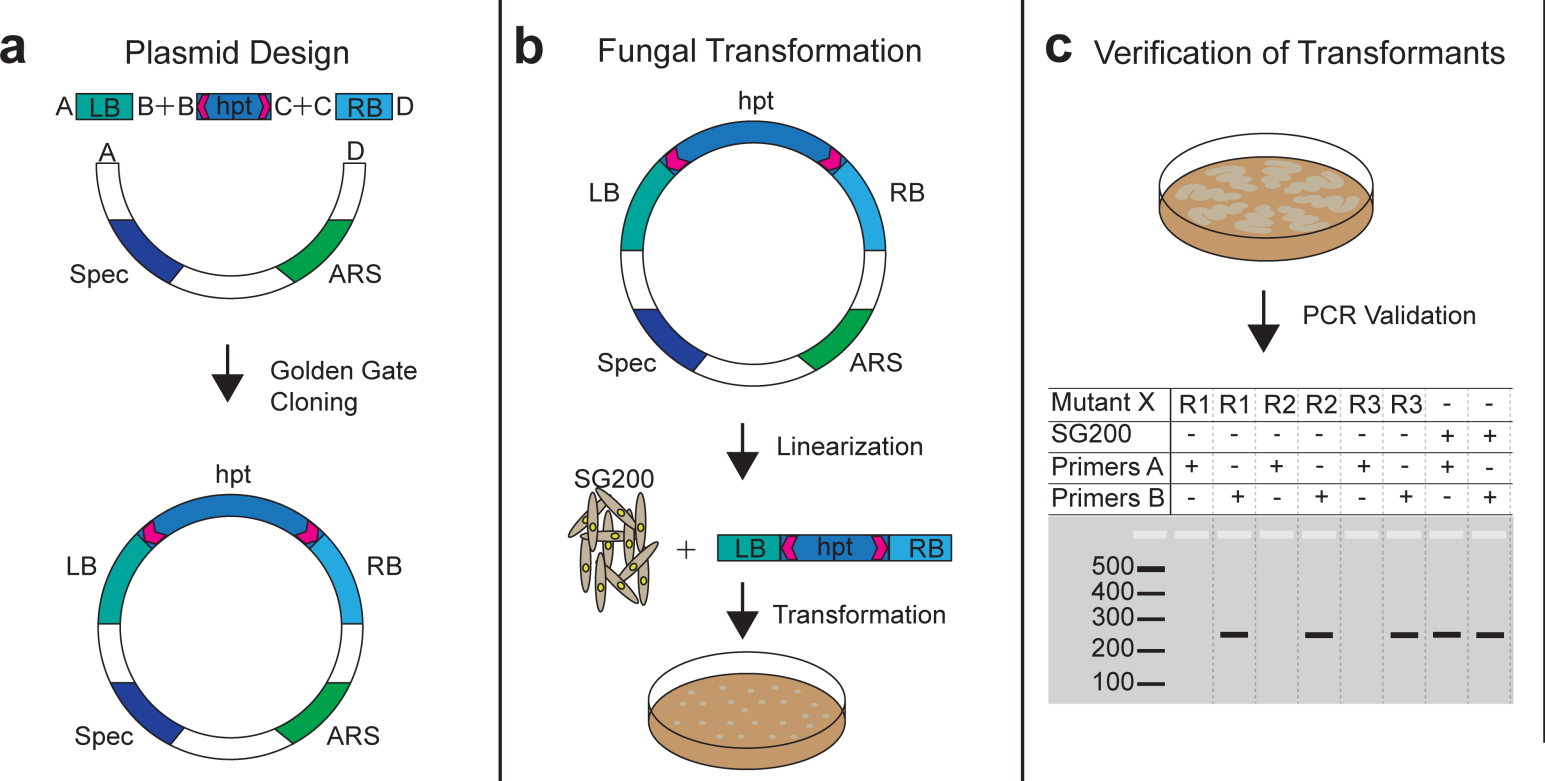
Design of deletion constructs and *U*. *maydis* insertional mutants. (**a**) Plasmid backbones containing a Spec and an ARS were combined with an hpt resistance cassette and specific borders (LB & RB) via Golden Gate Cloning [23]. The hpt resistance cassette is flanked by UPSs (magenta arrows). (**b**) Plasmids were linearized with *Asc*I and combined with haploid SG200 protoplasts. Transformants were selected on plates supplemented with hygromycin. (**c**) Schematic overview of PCR verification of transformants. Three independent fungal transformants were verified for each mutant locus via PCR. PCR products from primer-pair A targeting insertional mutant X was absent in positive transformants and detectable in SG200 control strains. A control primer-pair B gave a product in both insertional mutant X and SG200. ARS, autonomous replication sequence; hpt, hygromycin phosphotransferase; LB, left border; RB, right border; Spec, Spectinomycin resistance cassette; UPS, unique primer binding site.

We employed the smut fungus *U*. *maydis* as a model to establish iPool-Seq. We generated a Golden Gate cloning-compatible plasmid, which allows for recombination of multiple fragments in a single reaction [23]. To this end, we combined a hygromycin resistance cassette that is flanked by unique primer binding sites (UPSs) with the chromosomal up- and downstream regions (1,000 bp) of 195 predicted *U*. *maydis* effector genes (Fig 1A; S1 Table). Plasmids were linearized and transformed into *U*. *maydis* SG200 protoplasts for deletion of the putative virulence factors by homologous recombination (Fig 1B). For each of the insertion mutant constructs, we isolated 3 independent transformants and analyzed deletion events using PCR primers directed against the effector genes sequences. Absence of PCR products indicated successful deletions (Fig 1C). For each successful deletion, 3 independent transformant replicates were verified and stored separately, allowing for individual propagation to avoid growth competition prior to pooled infections. We performed 2 independent infections with pools containing the entire collection of 195 insertional mutants and established the iPool-Seq library preparation protocol (S2 Fig).

**Fig 2:**
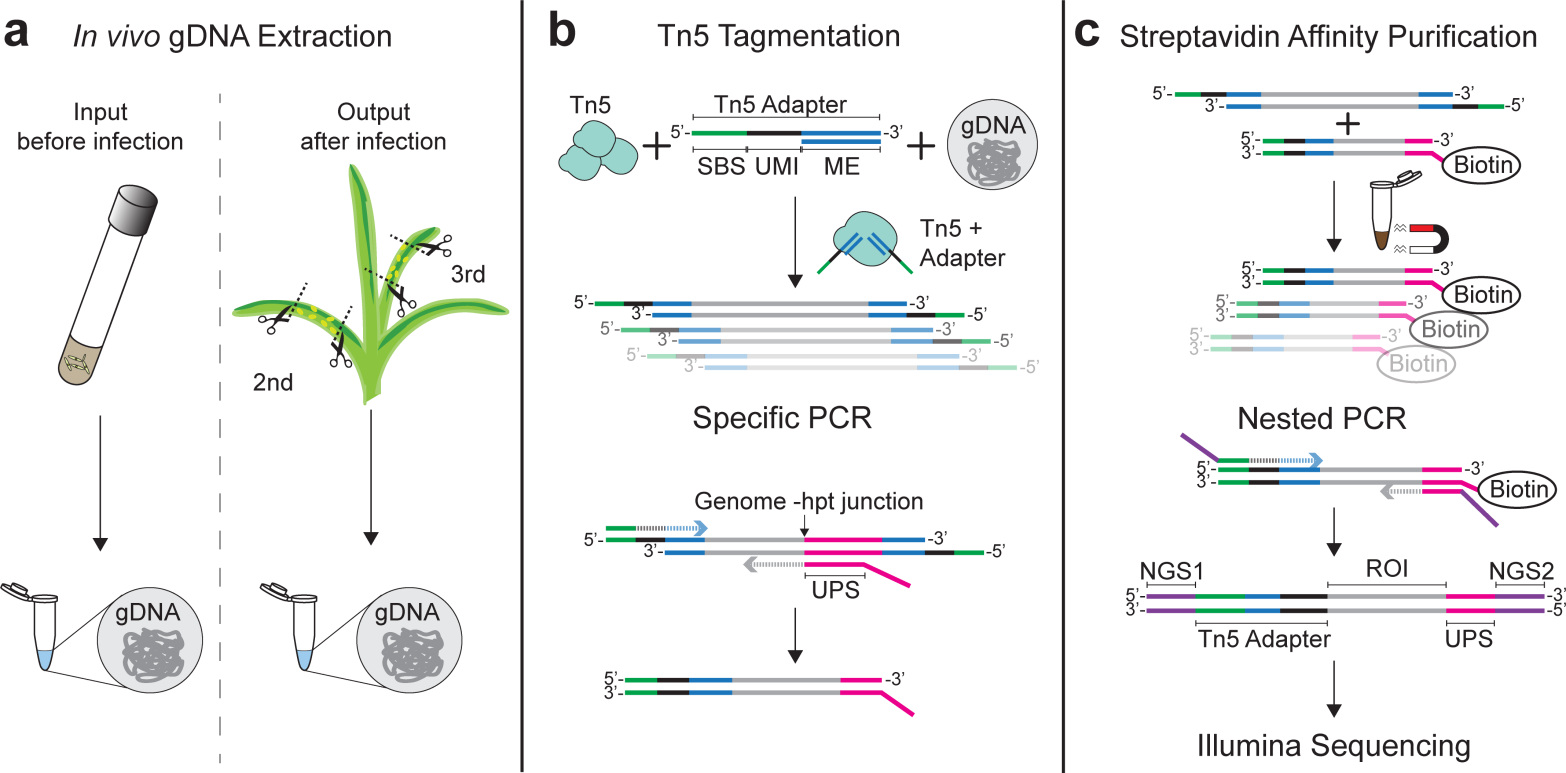
iPool-Seq library preparation workflow features tagmentation and UMIs. (**a**) Library preparation was carried out for the input mutant collection and for the output after infection. For the output, we harvested infected areas of the second and third maize leaves and isolated gDNA. (**b**) Extracted gDNA was fragmented with Tn5 Transposase loaded with custom adapters containing an SBS (green), 12-bp UMI, and Tn5 hyperactive MEs (blue). Genome–hpt resistance cassette junctions were PCR-amplified with biotinylated primers directed against UPSs (magenta) and adapter-specific primers directed at the SBS. (**c**) Biotinylated PCR products were streptavidin-affinity–purified and Illumina-compatible P5 (purple; NGS1) and P7 (purple; NGS2) ends were introduced by nested PCR. Final products were subjected to Illumina PE sequencing on a MiSeq platform. gDNA, genomic DNA; hpt, hygromycin phosphotransferase; iPool-Seq, insertion Pool-Sequencing ME, mosaic end; PE, paired-end; ROI, region of interest; SBS, sequencing primer binding site; UMI, unique molecular identifier; UPS, unique primer binding site.

For later comparison of mutant material abundance within the collection, iPool-Seq libraries were prepared from gDNA representing the mutant pool before infection (the input) and from infected tissues containing both maize and *U*. *maydis* genomes (the output, Fig 2A). To minimize the number of library preparation steps and conserve material, we replaced mechanical shearing of gDNA (requiring DNA-end repair, tailing, and adapter ligation steps) with Tn5-mediated tagmentation (Fig 2B) [21]. Although this approach yields a wider size range of DNA fragments, simultaneous DNA fragmentation and adapter ligation makes Tn5-mediated tagmentation preferable to DNA shearing approaches. We produced recombinant Tn5 transposase and adapted the published protocol to large gDNA inputs (S3 Fig) [21]. Furthermore, customized adapters for Tn5-mediated tagmentation were designed containing 12 bp unique molecular identifiers (UMIs) followed by a sequencing primer binding site (SBS; Fig 2B; S2 Table), which enables sequencing of UMIs using a custom-made first strand sequencing primer. Fragmented gDNA from pooled fungal infections of maize are not only highly diverse but fungal DNA content will certainly be underrepresented, making it necessary to efficiently enrich for insertion cassette junctions with genomic regions. To enrich for such junctions, the tagmentation-derived DNA fragments were amplified using specific adapter primers and biotinylated primers that bind to unique sequences at the distal ends of deletion cassettes (Fig 2B; S2 Table). Consequently, both genomic junctions of individual insertion cassettes were amplified, yielding biotinylated PCR products from all insertional mutants. Biotinylated PCR products were isolated using streptavidin-based affinity purification (Fig 2C) and Illumina-compatible adapters were introduced via nested PCR (S2 Table). Sequencing was performed on an Illumina MiSeq platform. In conclusion, we designed iPool-Seq to benefit from tagmentation, specific amplification, and streptavidin purification for efficient enrichment of ultra-rare genome deletion cassette junctions out of a highly diverse gDNA mixture.

### iPool-Seq facilitates the identification of fungal virulence factors

**Fig 3:**
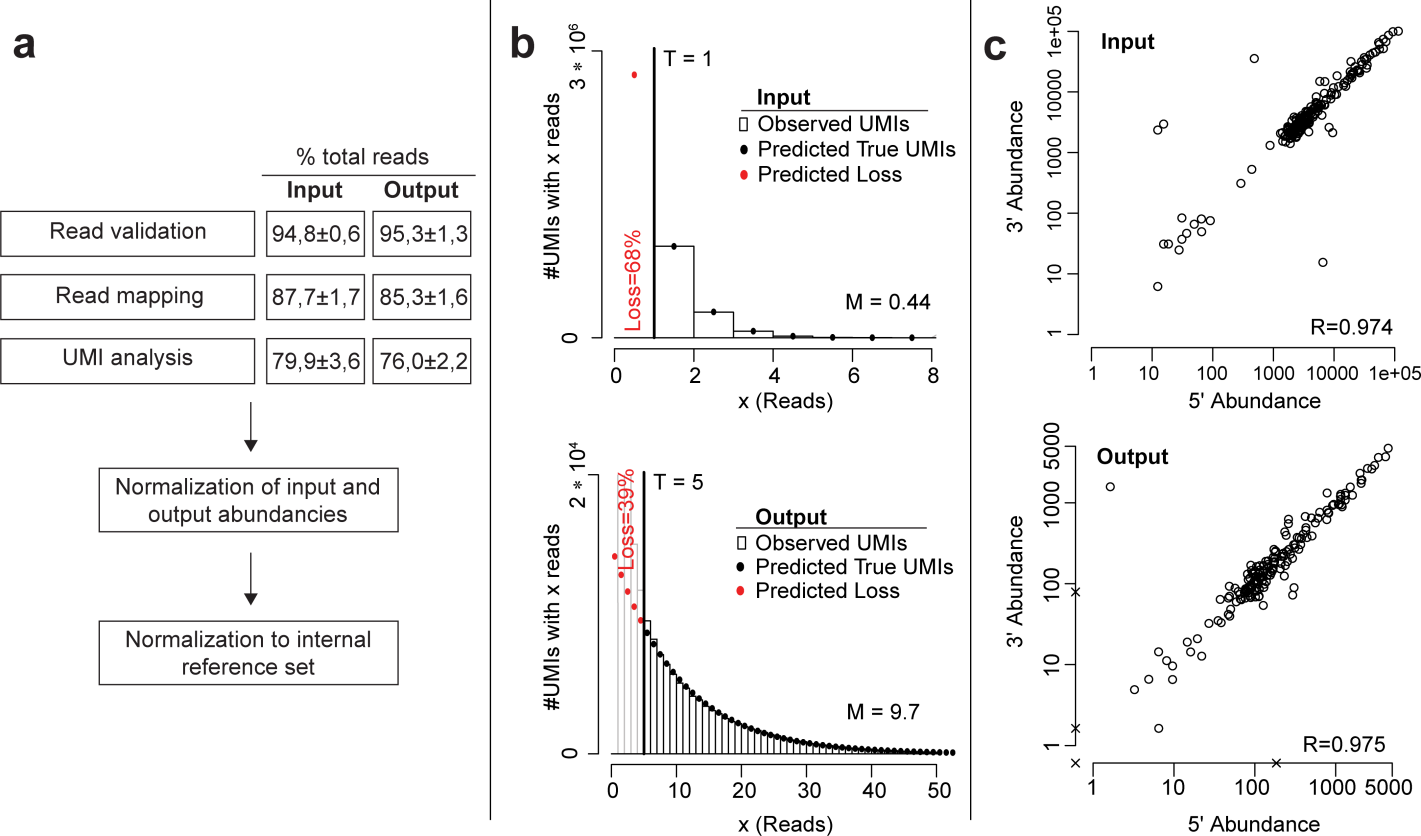
Quality control of iPool-Seq library. (**a**) Bioinformatic workflow of iPool-Seq analysis. Input and output read percentage after validation, mapping, and UMI analysis shows the mean ± SEM of 3 biological replicates and 2 independent infections. (**b**) Distribution of reads per individual UMI (bars) and model prediction (dots) over all insertional mutants of 1 representative replicate for input and output. Here, the error-correction threshold was set to 1 for the input and 5 for the output. Predicted true and lost UMIs are indicated. (**c**) Correlation plot of UMI counts for 5′- and 3′- genomic junctions of the hpt resistance cassette. One representative replicate of input and output is depicted. Each circle represents an insertional mutant. Missing up or downstream reads are marked with x. hpt, hygromycin phosphotransferase; iPool-Seq, insertion Pool-Sequencing; M, mean number of reads per UMI in the predicted distribution; R, correlation value; T, threshold; UMI, unique molecular identifier.

We infected maize in two independent experiments with three biological replicates of a pool of 195 verified insertional *U*. *maydis* mutants (S1 Table), resulting in six input and output libraries. The libraries were prepared as described above and sequenced on an Illumina MiSeq platform with paired-end (PE) sequencing. After read validation and read mapping, 87.7% ± 1.7% and 85.3% ± 1.6% of the obtained sequencing reads (input versus output, respectively) were mapped to *U*. *maydis* insertional mutation loci (Fig 3A; S1 Supporting methods).

To remove reads produced by PCR bias and which would affect quantitative evaluation of input and output reads, we collapsed all reads with highly similar UMIs to a single UMI count after sequencing. Based on the observed distribution of reads per UMI and comparison to a model prediction, we then set a library-specific read count threshold, removed UMIs with fewer reads than the threshold as likely PCR and sequencing artifacts, and corrected the number of remaining UMIs for the estimated loss of real UMIs (Fig 3B, S1 Supporting methods). After this UMI analysis, we retained 79.9% ± 3.6% and 76.0% ± 2.2% of initial reads from input and output for downstream analyses, respectively (Fig 3A).

The sequencing results indicated that three-fourths of all iPool-Seq reads were informational for insertion mutant abundance. Moreover, iPool-Seq generated similar amounts of valid reads from input- and output-derived gDNAs, indicating that yield performance was not diminished using gDNA derived from two organisms.

Since each inserted mutagenesis cassette has two junctions with neighbouring genomic regions, an unbiased library preparation should produce similar read numbers for up- and downstream junctions. We observed high correlation values (R) for all insertion mutants for the input and output samples, indicating that iPool-Seq is not suffering from considerable PCR biases during exponential amplification of DNA fragments containing mutagenesis cassette–genome junctions (Fig 3C).

**Fig 4:**
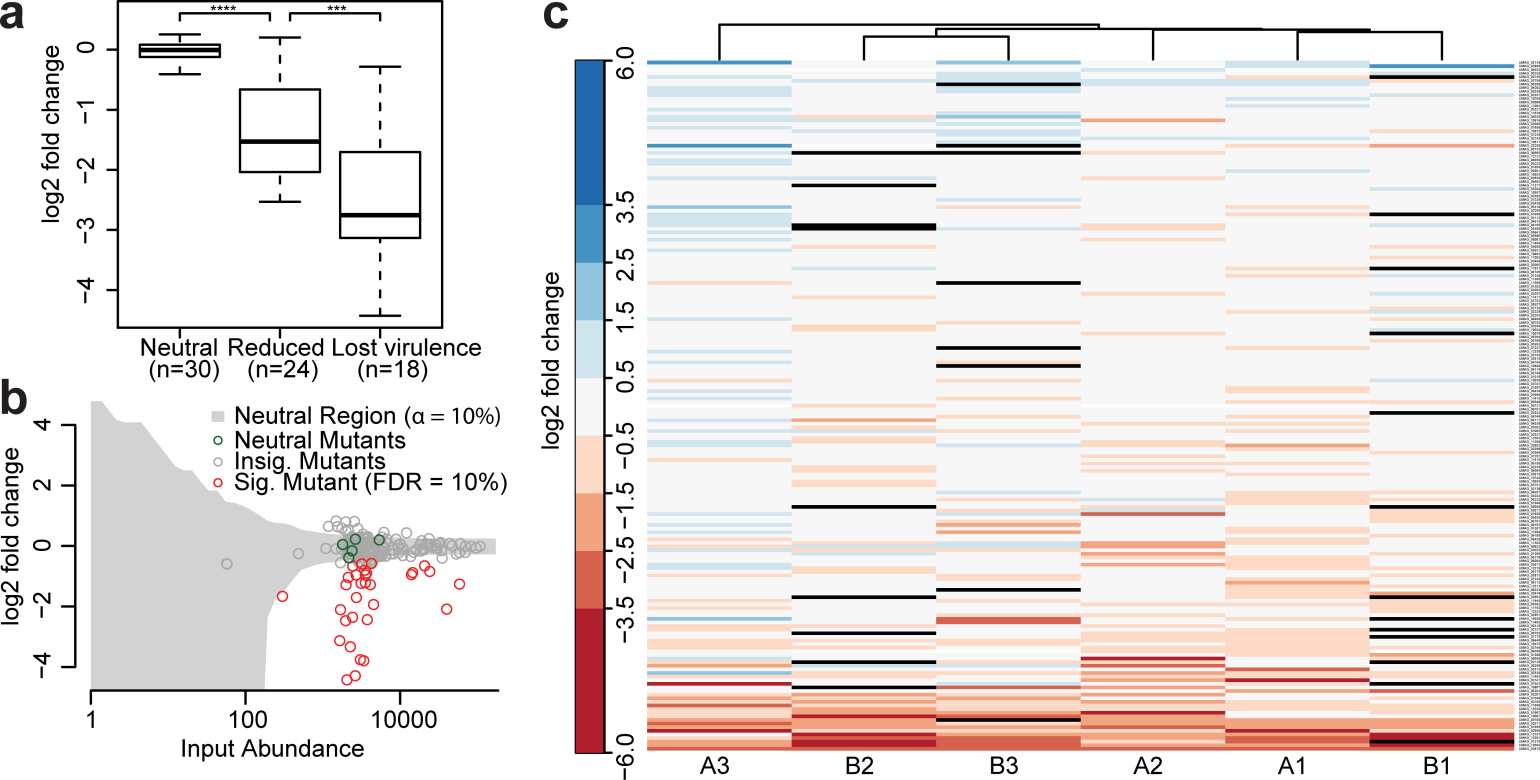
iPool-Seq identifies significantly depleted mutants after pooled infection. (**a**) Log_2_-fold changes between normalized output abundances and internal reference set for mutants with known phenotypes. *p*-Values were calculated with Mann–Whitney U tests. *p* = 5e^−9^ for neutral versus reduced and *p* = 3e^−4^ for reduced versus lost virulence with ***p < 0.001; **** *p* < 0.0001 (S3 Table). (**b**) Log_2_-fold change of output over input abundances for 1 representative replicate. Each circle represents 1 insertion mutant. Internal references are marked in green, significantly depleted in red (tested against reference set using negative binomial test; S1 Data; S1 Supporting methods), unaffected mutants in gray; Insig. area is also highlighted in gray. (**c**) Heatmap of log_2_-fold changes of input normalized UMI counts of all insertional mutants sorted by mean level of abundance. Infection A and B are two independent experiments and 1, 2, and 3 are three biological replicates, which were clustered according to similarity. Mutants without detectable reads in output libraries are displayed in black (S1 Data; S1 Supporting methods). FDR, false discovery rate; Insig., insignificant; iPool-Seq, insertion Pool-Sequencing; Sig., significant; UMI, unique molecular identifier.

To identify *U*. *maydis* virulence factors, we analyzed input and output reads for significantly depleted sequences from the pool of 195 insertion mutants. First, the read output of all insertional mutants was normalized to the corresponding input reads. Second, we defined an internal reference set of *U*. *maydis* mutant strains that do not have virulence phenotypes [18, 24] and whose output and input reads showed a neutral and linear relationship (Fig 4A, neutral; Fig 4B; S3 Table). Our collection contains additional mutants that were previously reported to be neutral. In these communications, neutral mutants formed symptoms with the same severity as the progenitor strain SG200. However, these observations did not provide any distinct information about quantitative growth defects of these mutants. Therefore, we constrained the neutral reference set to five mutants that displayed a reproducible neutral behavior in the iPool-Seq data (S1 Supporting methods).

We then calculated, for each mutant, the level of depletion from the output sample compared to the input and determined significance through normalization to the internal reference set. This resulted in the identification of a substantial proportion of sequences that were significantly depleted from the output libraries (Fig 4B, red circles; S1 Data). We analyzed this depleted sequence set for known virulence factors and identified Pep1, Pit2, and Stp1 (UMAG_01987, UMAG_01375, and UMAG_02475) [25–27] as known essential virulence factors of *U*. *maydis* (Fig 4A, lost virulence). In addition, we found the previously described virulence factors ApB73 (UMAG_02011) [28] and Fer1 (UMAG_00105) [29] among the less depleted and reduced candidate sequences (Fig 4A, reduced). Two other mutants (UMAG_06223 and UMAG_02239), for which minor defects in disease symptom induction had been reported previously, were not significantly depleted in the iPool-seq results and one mutant (UMAG_12313) previously reported to be unaffected in virulence showed a weak but significant reduction in our iPool-seq approach (S4 Table) [24]. In summary, iPool-Seq results largely overlap with previously reported symptom scoring data for characterized virulence factors (S4 Table). It is also sensitive, as not only apathogenic but also reduced virulence factor mutants were identified. Importantly, analysis of the depleted sequence set yielded 23 fungal mutants that are potential novel virulence factors of *U*. *maydis* (Fig 4C; S4 Table).

In contrast to the identification of depleted mutant sequences, we did not identify sequences that were reproducibly enriched in all biological replicates, indicating that none of the fungal mutants tested conferred enhanced virulence to *U*. *maydis* on the tested host accession Early Golden Bantam (EGB; Fig 4C).

We next modeled the performance of iPool-Seq on a high-throughput mutant library of *U*. *maydis* (S9 Fig, S1 Supporting methods). To this end, we used the following parameters: 1) 20,000 insertion mutants were chosen cover the approximately 20-MB genome of *U*. *maydis* with approximately 1,000 bp average distance of insertion sites. 2) During maize colonization, approximately 1,500 of the approximately 6,900 *U*. *maydis* genes are transcriptionally up-regulated—and we showed that about 14% of all mutants from our library contributed to virulence (Fig 4C; S4 Table) [18, 30]. Based on these observations, we extrapolate that approximately 3% of all *U*. *maydis* genes are likely to be involved in virulence. 3) We showed with iPool-Seq that known reduced virulence factors of *U*. *maydis* had a mean logarithmic fold change of −1.53 and known essential virulence factors of −2.75 in comparison to the neutral reference set, respectively (Fig 4A). Due to a lack of data, the model does not take into account the number of unsuccessful infection events on the host plant but assumes 100% infection rate for each individual of a neutral mutant strain.

The model resulted in 40 (for essential virulence factors) and, respectively, 100 (for weak virulence factors) detected individuals necessary for each mutant in the input samples to identify virulence factors with 99% sensitivity. Based on observed average of approximately 10 reads per UMI (Fig 3B) and due to the insertion flank sequencing efficiency of at least 75% (Fig 3A), the required sequencing depth would be 26 Mio reads (20,000·100·10·1,33 = 26,600,000) per library. This suggests that the iPool-Seq technology can be used for large scale mutant screens in *U*. *maydis* and similar systems.

### Validation of novel essential *U*. *maydis* virulence factors

**Fig 5:**
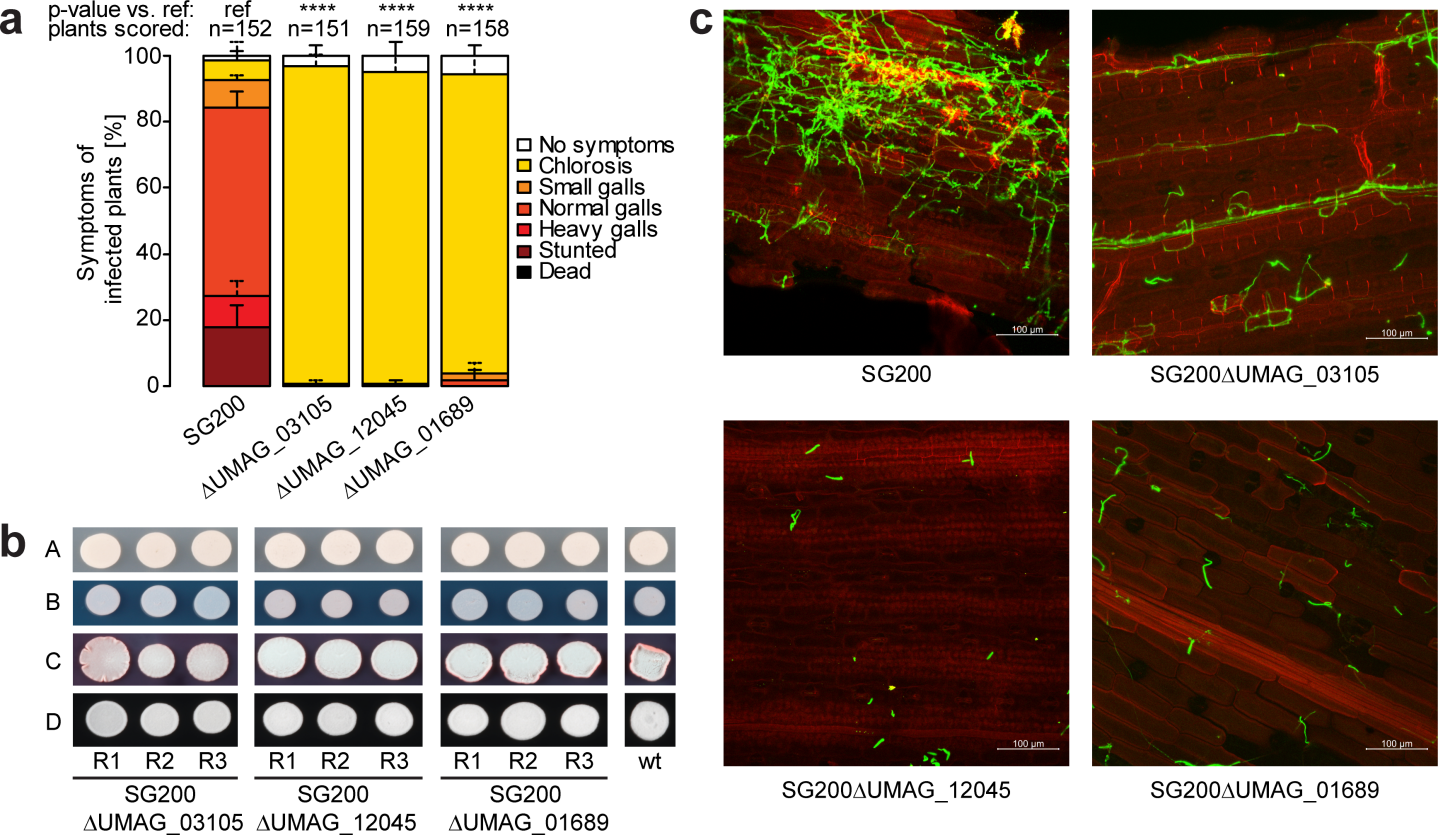
Virulence factor mutants identified by iPool-Seq cause reduced disease symptoms on maize. (**a**) Disease rating of insertional mutant strains 7 dpi. Mean standard deviation of relative counts from 3 replicates are displayed. Only positive error bars are shown. *p*-Values were calculated by Fishers exact test. Multiple testing correction was done by Benjamini-Hochberg algorithm. **** *p* < 0.0001. (S2 Data) (**b**) Growth assay of insertion mutants on (A) Cm-medium, or Cm-medium supplemented with (B) 75 μg/mL Calcofluor (cell wall stress), (C) 45 μg/mL Congo red (cell wall stress), and (D) Charcoal (b-filament inducing). (**c**) Confocal microscopy of maize infected with indicated insertional mutant strains 7 dpi. Infected plant tissue was stained with propidium iodide (red) and fungal hyphae with lectin binding WGA-AF488 (green). One representative picture of 9 infected plants is shown. Cm-medium, control Complete medium; dpi, days post infection; iPool-Seq, insertion Pool-Sequencing; ref, reference; wt, wild-type.

To validate the 23 potential virulence factors identified by iPool-Seq, we chose three top candidates and tested their effects on virulence using individual infection assays. We observed a severe loss of *U*. *maydis* virulence upon infection of plants with fungi carrying these mutations. Whereas the wild-type progenitor strain SG200 produced galls on infected maize, all three mutant strains failed to form galls, indicating that they are essential for fungal virulence (Fig 5A). This effect was specifically due to virulence, as growth assays under stress-inducing conditions showed no difference between these mutant strains and SG2000 (Fig 5B). Using confocal microscopy on infected plants, we observed that mutant strains were severely impaired in colonizing maize leaf tissues (Fig 5C). Our combined results show that iPool-Seq facilitates the identification of essential factors for *U*. *maydis* virulence. Furthermore, the streamlined library preparation of iPool-Seq should make the method widely applicable for identifying unknown virulence factors in complex biological systems, such as in vivo infected tissues.

## Discussion

Pooled mutant screens have proven to be very powerful tools to uncover individual genes affecting particular phenotypes in a time- and cost-effective fashion. Positive selection screens usually lead to limited numbers of individual surviving cells that are easily identifiable by a combination of restriction enzyme digests, inverse PCR, and sequencing. Negative selection screens rely on the survival of most analyzed cells, making it necessary to devise methodology that allows comparing the presence/absence of genetic information before and after selection. To tackle the later challenge, several insertional mutagenesis approaches have been developed [31]. Although successful in bacterial systems for the elucidation of virulence factors [5, 13, 32], such insertion mutant approaches were not widely used in eukaryotic systems, mainly because of unresolved technical issues such as low sensitivity and system-intrinsic limitations (for example, genome ploidy, lifestyle of the investigated model system).

Here, we introduce iPool-Seq as a versatile and highly sensitive method for the analysis of insertion mutant pools before and after selection, enabling both negative and positive mutant selection screens in complex eukaryotic systems including the analysis of host–pathogen interactions. We used iPool-Seq to examine virulence factors from a defined set of mutants of the crop fungus *U*. *maydis*, both confirming known factors and identifying novel ones. From the predicted mutant collection we used, most mutants were not significantly depleted from the output reads, indicating no function in virulence for the underlying genes. However, the role of some factors could be difficult to decipher, for example, because their action could be covered by functional redundancy of other virulence factors. Although we infected insertion mutants in dense pools, depleted insertional mutants appeared not to be affected by in trans complementation, by using the secreted factors of neighbouring fungal cells for example. Nevertheless, it cannot be excluded that, for certain gene products, in trans complementation could occur and mask the virulence defect of the respective mutant in a pooled infection setup. In conclusion, negative depletion screens have limitations to decipher redundancy and potential in trans complementation of virulence factors. In addition, we did not identify significantly enriched mutants in the iPool-Seq analysis of the mutant collection. A significant enrichment of output reads would indicate the loss of a negative regulator of virulence. A possible reason that we did not find enrichment could be our choice of the maize accession, EGB, which is highly susceptible to the *U*. *maydis* strain SG200.

Microscopy of *U*. *maydis* strain SG200 infecting maize tissue implies that many cells fail to penetrate the host [28]. In very complex insertion mutant libraries, this large individual failure rate could lead to the loss of mutants that lack any real defect in virulence. Therefore, for genome-wide virulence maps of *U*. *maydis* and similar biotrophic pathogens, the size of the insertion mutant pool must be individually adapted to the infection rate of the respective pathogen. To overcome this problem, genome-wide screens might need to be performed in subpools, as it has been done in a previous study with the fungal pathogen *Cryptococcus neoformans* [11].

iPool-Seq uses insertion cassette–specific primers to amplify the genomic insertion junctions from a mutant pool [17]. Additionally, iPool-Seq enriches for PCR products by using biotin/streptavidin interaction, an approach that has previously been used in bacterial transposon integration site identification methods such as high-throughput insertion tracking by deep sequencing (HITS) [5]. Importantly, UMIs in the adapter primer allow in silico elimination of PCR biases. The unique barcode identifiers additionally overcome cluster position identification problems during Illumina sequencing that would otherwise occur when the first bases from the insertion flank would otherwise be identical for all mutant loci. Dark cycle sequencing, as used in Quantitative insertion-site sequencing (QIseq) for example, is therefore unnecessary [17].

iPool-Seq was established using a defined insertion mutant collection of *U*. *maydis*. However, the technology can be adapted to any insertion mutant collection, such as transposon or *A*. *tumefaciens*-derived T-DNA libraries [33, 34]. The modeling of the iPool-Seq sensitivity indicates that iPool-Seq meets all premises to work for high-throughput. Therefore, iPool-Seq promises to be a versatile technology for reanalysis of existing knock-in, activation-tagging, or transposon-insertion libraries, dramatically reducing labor costs for selection screens when compared to classical scoring approaches. Additionally, the relatively low costs of iPool-Seq for broad screens could also foster research in less funded emerging model systems. Due to the strong enrichment of insertion gene cassettes, the sequencing depth and costs of iPool-Seq are low. Thus, this technology will enable researchers to test diverse new selection criteria to efficiently build genotype–phenotype relationships. This will help to fill the knowledge gap that is currently still hampering research as exemplified for the well annotated *U*. *maydis* genome with 6,786 protein-encoding genes, of which 41.5% are in the category unknown [35]. Moreover, even if genes are annotated, their involvement in various biological processes might, simply, not yet be known.

From the candidate virulence factors that we identified with iPool-Seq, we chose 3 for verification and confirmed their virulence defect by classical scoring of disease symptoms. However, the assessment of disease symptoms is indirect, and discrepancies between the two methods might occur for other novel virulence factors. We speculate that the *U*. *maydis* genome encodes virulence factors whose mutants show reduced proliferation but still cause full disease symptoms based on qualitative measures. In line with this, the iPool-Seq data did not show significant depletions for two mutants that were previously reported with mild defects in symptom induction [24]. In contrast to these disease ratings, iPool-Seq has the potential to identify virulence factors that do not have an obvious effect on symptom formation on a genome-wide level.

In summary, we have demonstrated the functional genomic technology iPool-Seq by identifying both known and novel virulence factors from pooled infection assays of a biotrophic fungus within a complex host background. iPool-Seq is therefore a sensitive in vivo tool for researchers to help fill the genotype–phenotype gap in the post-genomic era.

## Methods

### Vector construction and insertional mutant generation

For all DNA manipulation we used *Escherichia coli* Mach1 (Thermo Fisher Scientific). The vector backbone for the generation of the mutant collection is based on pGBKT7 (Clontech Laboratories). We replaced kanamycin resistance with a spectinomycin resistance cassette and removed internal *Sap*I, *Bsa*I, *BsmB*I, and *Bbs*I restriction sites by direct mutagenesis from a derivative of the original vector, respectively [36]. The hygromycin resistance marker originates from vector pHwtFRT [37]; and *Sap*I, *Bsa*I, *BsmB*I, and *Bbs*I restriction sites were removed by site-directed mutagenesis. Moreover, we elongated the hygromycin cassette with a UPS on the 5′- and 3′-end (5′-TCGCCACAGGATACCACAGGACATCTGGGATATC and 3′-GCCACTCACGCCACAGGATACCACAGGACATCTGGGATATC; UPS is underlined). In detail, for each mutant locus we amplified 1,000 bp up- and downstream borders from *U*. *maydis* gDNA with standard molecular cloning procedures [38] and combined them with the modified hygromycin-selectable marker cassette flanked with UPS (Fig 2; S2 Table) and the plasmid backbone. Depending on the occurrence of internal restriction sites, we used either *Sap*I, *Bsa*I, *BsmB*I, or *Bbs*I restriction sites (ordered by priority of choice) for Golden Gate cloning [23]. Constructs were verified by Sanger sequencing and subsequently transformed into the haploid solopathogenic strain SG200 of *U*. *maydis* as previously described [18, 39, 40]. Transformants were verified by direct PCR: single mutants were grown in YepsLight (0.4% yeast extract, 0.4% peptone and 2% sucrose) liquid medium at 28°C with shaking at 200 rpm in 48-well plates overnight. The next day, 100 μL overnight culture was pelleted and resuspended in 20 μL 0.02 M NaOH. 1 μL was then utilized for a direct PCR reaction with a primer pair directed against the replaced gene. As a positive control, a primer pair binding to another mutant locus was used. Subsequently, we isolated gDNA from at least 4 PCR positive strains and repeated the direct PCR using 1 μL of 1:10 diluted gDNA as a template. All primer pairs used for the verification of deletion strains produced PCR products from a gDNA template from the progenitor strain SG200. Three independently verified *U*. *maydis* insertional mutants were preserved at −80°C in PD liquid supplemented with 50% glycerol.

### Growth conditions and pooled infection

For each mutant collection pool replicate we infected at least 100 plants of maize variety EGB (Olds Seeds, Madison, WI, USA). Seedlings were grown under a 14-hour/10-hour light/dark cycle at 28°C/20°C in plant growth chambers and infected 7 days after potting. *U*. *maydis* mutant strains were grown individually on selective PD plates supplemented with 200 μg/mL hygromycin for 2–3 days at 28°C. Subsequently, for each mutant strain, 1 mL YepsLight (0.4% yeast extract, 0.4% peptone and 2% sucrose) liquid preculture was inoculated in 48-well plates and grown at 28°C overnight with shaking at 200 rpm. For main cultures, precultures were diluted 1:2,000 in 3 mL YepsLight in test tubes and grown at 28°C with shaking at 200 rpm overnight. After 14–16 hours, the main cultures of all mutants were adjusted to an OD_600_ of 3 and mixed in equal amounts. The mutant pool was pelleted at 2,000 x g for 10 minutes and resuspended in sterile water. 250 μL of the mutant pool was infected in each maize seedling with a syringe. After 7 days, infected areas from the second and third leaves were harvested, ground to a fine powder in liquid nitrogen, and preserved at −80°C until iPool-Seq library preparation.

### iPool-Seq library preparation

For output gDNA extraction, 0.75–1 g of infected plant powder was supplemented with 2 mLLysis buffer (10 mM Tris, pH 8; 100 mM NaCl; 1 mM EDTA; 2% Triton X 100 [v/v]; 1% SDS [w/v]), 2.5 mL TE-buffer equilibrated phenol, chloroform, and isoamyl alcohol (25:24:1, pH 7.5–8, Carl Roth) and 100 μL sterile glass beads (450–600 μM, B.Braun) in a 7-mL Precellys tube. The material was processed for 20 seconds at 4,500 rpm with a Precellys evolution bead mill (Bertin). The debris was pelleted at 17,000 x g for 15 minutes, and 2 mL supernatant was added to 2.2 mL Isopropanol. The precipitated gDNA was washed with 1 mL 80% EtOH and eluted in 150 μL or 200 μL TE supplemented with RNAse A (20 μg/mL, Thermo Fisher Scientific). For input gDNA extraction, gDNA was extracted from 2 mL of insertional mutant pool as previously described [41]. gDNA concentrations were determined with PicoGreen (Thermo Fisher Scientific). Tn5 fragmentation of a total of 10 μg gDNA for output and 1 μg gDNA for the input was adapted from [20], and performed as follows [21]: We combined 1 μg gDNA per 20 μL reaction with Tn5 transposase (150 ng/μL f.c.) preloaded with 25-μM adapters in 1x TAPS buffer (50 mM TAPS-NaOH, 25 mM MgCl2, 50% v/v DMF, pH 8.5 at 25°C) and incubated the reaction mix in a thermocycler at 55°C for 10 minutes. We purified each reaction mix with a 1:1 ratio of Agencourt AMPure XP beads (Beckman Coulter) according to the manufacturer’s protocol and performed PCR with Phusion polymerase (New England Biolabs) using an adapter specific forward primer and a biotinylated insertion specific primer from 250 ng fragmented gDNA (denaturation for 15 seconds at 95°C, annealing for 15 seconds at 65°C, elongation for 30 seconds at 72°C; repeated for 15 cycles; 1 minute final elongation). We pooled all PCRs of the same sample and purified 1/5 with Agencourt AMPure XP beads (ratio 1:1; Beckman Coulter). The PCR amplicons eluted from each sample were split into 4 PCR reactions and amplified with nested primers to add Illumina compatible P5 and P7 ends (15 cycles, with 65°C annealing temperature and 30 seconds elongation at 72°C). The final PCR products were purified with Agencourt AMPure XP beads in a 1:1 ratio. The average fragment size was measured on a fragment analyzer (Advanced Analytical Technologies, Inc.) and library quality was controlled with qPCR. Illumina Sequencing was performed on a MiSeq platform with 75 PE conditions. We used a custom designed forward sequencing primer and the standard Illumina primers for reverse and index sequencing (S2 Table).

### Confirmation of iPool-Seq candidate virulence factors

We confirmed the results of iPool-Seq for 3 candidate genes with individual infection assays, microscopy, and in vitro growth assays. The infection assay was performed as previously described [18]. In summary, for each insertional mutant, 3 replicates of *U*. *maydis* were grown overnight in YepsLight liquid medium (0.4% yeast extract, 0.4% peptone and 2% sucrose) with 200 rpm agitation to an OD_600_ of 0.6–1 and adjusted to an OD_600_ of 1 in sterile water. We syringe-infected 7-day-old maize seedlings of the variety EGB with approximately 250 μL fungal suspension per plant. Symptoms were scored 7 days post infection (dpi) according to the published protocol [18]. Fungal leaf colonization was assessed 7 dpi via microscopy. Fungal hyphae were stained with WGA-AF488 (Thermo Fisher Scientific) and plant cell walls with propidium iodide (Sigma-Aldrich) as previously described [28]. Confocal microscopy was performed with the following settings: We utilized an LSM780 Axio Observer confocal laser scanning microscope with an LD LCI Plan-Apochromat 25x/0.8 Imm Corr DIC M27 objective (Zeiss, Jena, Germany). WGA-AF488 was excited at 488 nm and detected at 500–540 nm; propidium iodide was excited at 561 nm and detected at 580–660 nm.

### Bioinformatic analysis

For each sequenced library, adapter read-throughs were removed from the raw Illumina reads, UMIs were extracted and stored separately, and the reads (lacking UMIs) were mapped to the *U*. *maydis* reference genome [18] using NextGenMap [42]^.^ The reads mapping to each flank (5' and 3') of each insertional mutant were grouped by UMI, and highly similar UMIs were merged to correct for sequencing errors [43]. UMIs with fewer reads than the error-correction threshold were removed as likely artifacts, and the number of surviving (and thus likely true) UMIs for each gene and flank were counted. To correct for biases caused by different detection losses (i.e., # undetected genomes/# total genomes) between mutants and flanks, the mutant- and flank-specific losses were estimated from the observed mutant- and flank-specific distributions of reads per UMI (S1 Supporting methods) using the TRUmiCount algorithm (see S1 Supporting methods for details) [44]. To discern stochastic fluctuations from knockout phenotypes, the number of true UMIs detected in the output pool for neutral insertional mutants were assumed to follow a negative binomial distribution with mean $\mu_{m}=\lambda \cdot n_{m}^{\text{in}}\cdot \left(1-l_{m}^{\text{out}}\right)/\left(1-l_{m}^{\text{in}}\right)$ and (inverse) overdispersion parameter $r_{m}=n_{m}^{\text{in}}/\left(1+d\cdot n_{m}^{\text{in}}\right)$. Briefly, a neutral mutant *m*’s expected UMI count in the output pool thus depends on (1) the number $n_{m}^{\text{in}}$ of detected UMIs in the input pool, (2) the estimated losses $l_{m}^{\text{out}}$ and $l_{m}^{\text{in}}$ for the output and input pool, and (3) a mutant-independent normalization factor *λ* to account for differences in total genome count between input and output samples. The sources of overdispersion of the output counts are (4) the (Poissonian) sampling uncertainty of the input pool counts $n_{m}^{\text{in}}$, and (5) random fluctuations of fungus proliferation accounted for by the mutant-independent parameter *d*. For each output pool, parameters *λ* and *d* were estimated (see S1 Supporting methods for details) by fitting the model to a reference set of presumed neutral mutants (S3 Table), 2 one-sided *p*-values for the significance of depletion (respectively, enrichment) compared to the reference set were computed for each insertional mutant and transformed to q-values to control for the false discovery rate (FDR) [45]. Undetected insertional mutants (i.e., with zero UMIs) in input pools were excluded from the analysis of the corresponding output pools. Undetected insertional mutants in output pools were not assigned *p*- or q-values.

To quantify the change in virulence of an insertional mutant, its abundance in the output was first normalized to its abundance in the input (thus assuming independent fates of the individuals in the input). Then, the log_2_-fold change between its normalized output abundance and the normalized output abundance of the internal reference set was computed (see S1 Supporting methods for details). Further details on the modeling can be found in S1 Supporting methods.

## Supporting information

S1 Data q-Values of *U*. *maydis* mutant strains. (XLSX)

S2 Data Symptom rating of mutant strains. (XLSX)

S1 Fig Workflow of pooled infection of maize. For each replicate of the *U*. *maydis* mutant collection, at least 100 maize plants of the accession EGB were potted. Mutants were grown on selective plates for 2–3 days. From plates, precultures were inoculated and grown ON. The precultures were used for inoculation of the main cultures to avoid dead material in the infection pool. All main cultures were pooled with equal amounts that were adjusted to the same optical density and infected in 7-day old maize seedlings with a syringe. Infected areas of the second and third leaf of each plant were harvested 7 days after the infection. All 3 biological replicates of the mutant collection were processed in 14 days. EGB, Early Golden Bantam; ON, overnight.
(TIF)

S2 Fig Tn5 fragmentation of gDNA with modified adapters. Recombinantly produced hyperactive Tn5 was tested with standard Tn5-ME-A and custom UMI-ME-A on 1 μg gDNA of *U*. *maydis*-infected maize tissue with indicated concentrations. gDNA; genomic DNA; In, Input; M, Marker 1 kb-ladder (Thermo Scientific); ME, mosaic end; Tn5-ME-A, Tn5-ME-Adapter; UMI-ME-A, UMI-ME-adapter.
(TIF)

S3 Fig Sensitivity of iPool-Seq. Estimated sensitivity of iPool-Seq for a genome-wide library of *U*. *maydis* mutants. Model shows for different (1 up to 100) mutant copies detected in the input sample for the sensitivity of virulence factor detection. Depicted model curves are given assuming 3% of all mutants have a reduced virulence of log2(FC) −1.53 and log2(FC) of −2.75, respectively, and the other 97% are neutral in respect to virulence. The sensitivity reaches 99% at 40 detected mutants (lost virulence) and 100 detected mutants (reduced virulence), respectively. FC, fold change; iPool-Seq, insertion Pool-Sequence.
(TIF)

S1 Software iPool-Seq analysis pipeline. iPool-Seq, insertion Pool-Sequencing.
(TGZ)

S1 Supporting methods iPool-Seq analysis pipeline description. iPool-Seq, insertion Pool-Sequencing.
(PDF)

S1 Table *U*. *maydis* genes targeted for insertional mutagenesis. (XLSX)

S2 Table Key primers used in this study. (XLSX)

S3 Table *U*. *maydis* mutants used for the internal reference set. (XLSX)

S4 Table Significantly depleted *U*. *maydis* mutants identified by iPool-Seq. iPool-Seq, insertion Pool-Sequencing.
(XLSX)

## Abbreviations

ARS: autonomous replication sequence
Cm-medium: control Complete medium
dpi: days post infection
EGB: Early Golden Bantam
FDR: false discovery rate
gDNA: genomic DNA
hpt: hygromycin phosphotransferase
HITS: high-throughput insertion tracking by deep sequencing
iPool-Seq: insertion Pool-Sequencing
LB: left border
ME: mosaic end
NGS: next-generation sequencing
PE: paired-end
RB: right border
ROI: region of interest
SBS: sequencing primer binding site
UMI: unique molecular identifier
UPS: unique primer binding site
wt: wild-type
