# Supplementary figures and images for: In vivo insertion pool sequencing identifies virulence factors in a complex fungal–host interaction
Source: PLoS Biol. 2018 Apr 23;16(4):e2005129. doi: 10.1371/journal.pbio.2005129 (PMC5912717; doi:10.1371/journal.pbio.2005129)

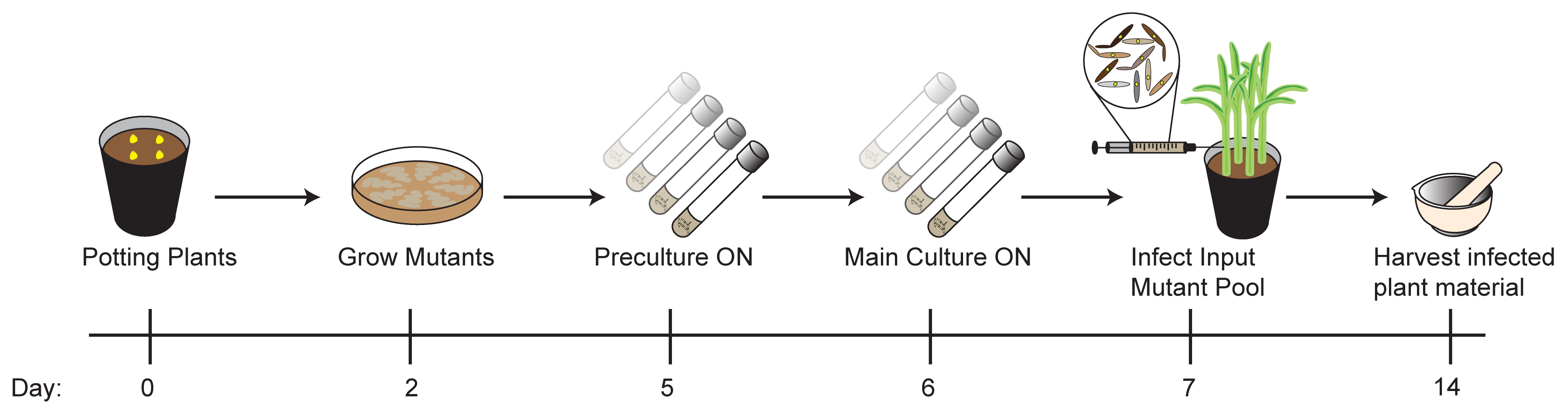

Supplement: S1 Fig — For each replicate of the U. maydis mutant collection, at least 100 maize plants of the accession EGB were potted. Mutants were grown on selective plates for 2–3 days. From plates, precultures were inoculated and grown ON. The precultures were used for inoculation of the main cultures to avoid dead material in the infection pool. All main cultures were pooled with equal amounts that were adjusted to the same optical density and infected in 7-day old maize seedlings with a syringe. Infected areas of the second and third leaf of each plant were harvested 7 days after the infection. All 3 biological replicates of the mutant collection were processed in 14 days. EGB, Early Golden Bantam; ON, overnight. (TIF) [file pbio.2005129.s003.tif]

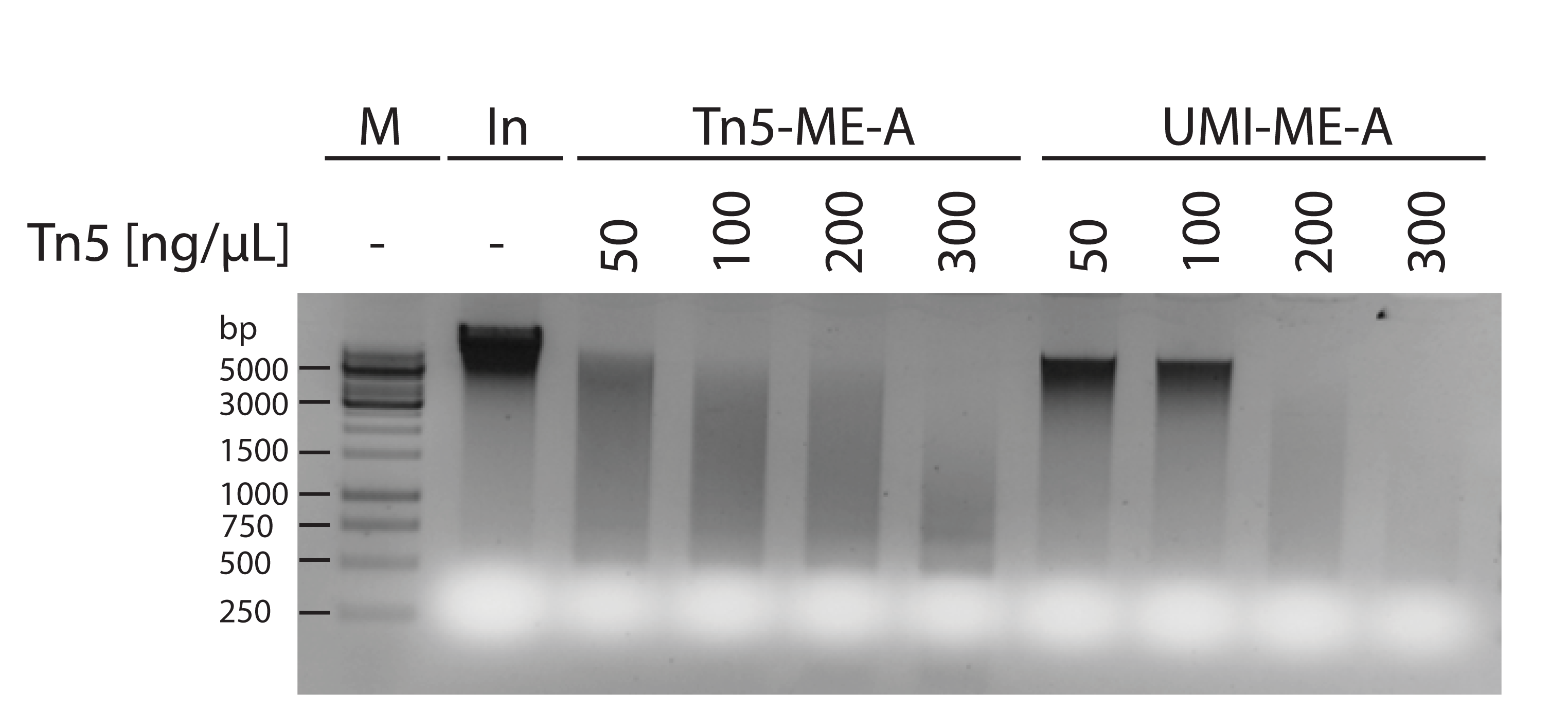

Supplement: S2 Fig — Recombinantly produced hyperactive Tn5 was tested with standard Tn5-ME-A and custom UMI-ME-A on 1 μg gDNA of U. maydis-infected maize tissue with indicated concentrations. gDNA; genomic DNA; In, Input; M, Marker 1 kb-ladder (Thermo Scientific); ME, mosaic end; Tn5-ME-A, Tn5-ME-Adapter; UMI-ME-A, UMI-ME-adapter. (TIF) [file pbio.2005129.s004.tif]

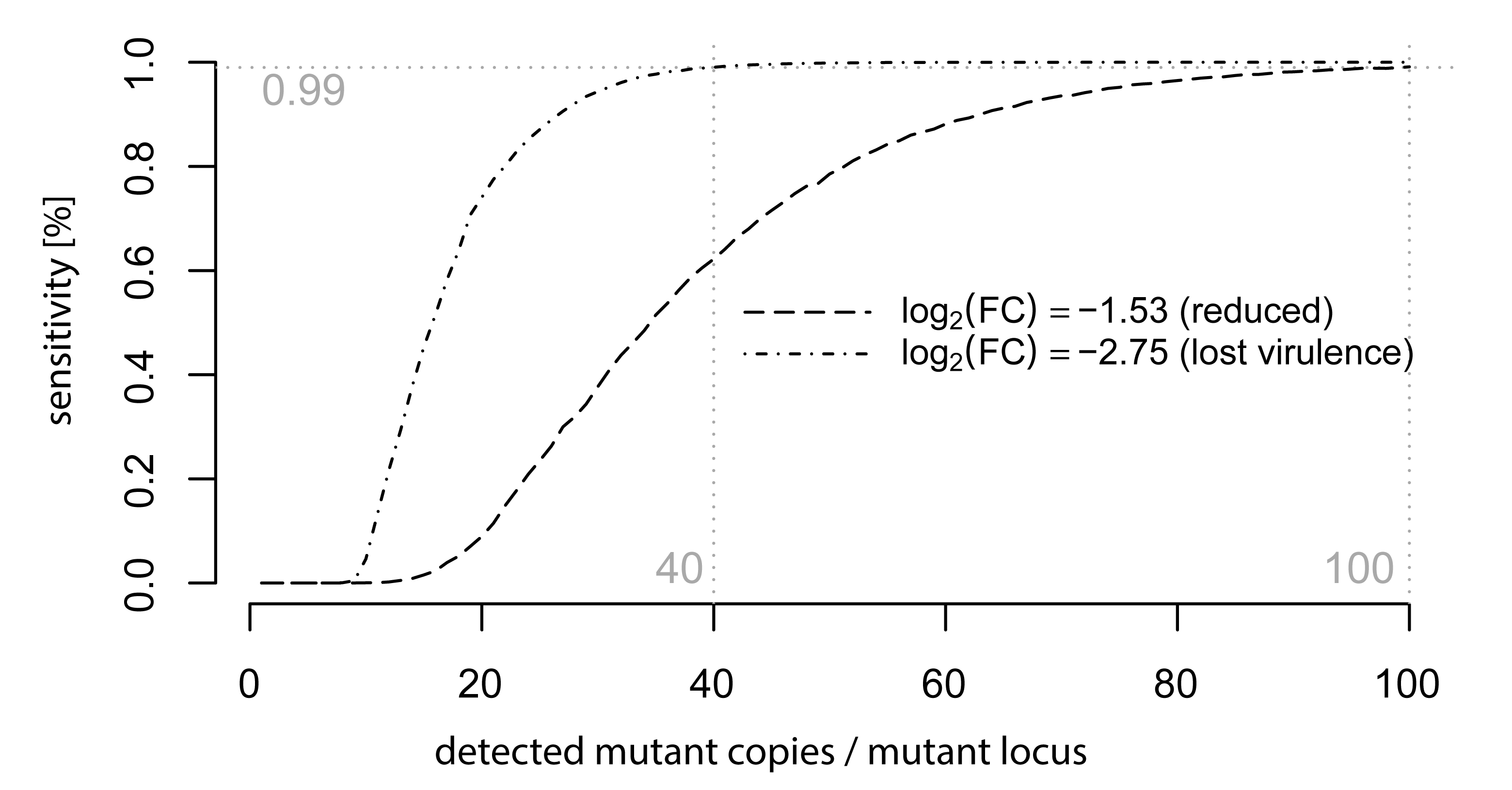

Supplement: S3 Fig — Estimated sensitivity of iPool-Seq for a genome-wide library of U. maydis mutants. Model shows for different (1 up to 100) mutant copies detected in the input sample for the sensitivity of virulence factor detection. Depicted model curves are given assuming 3% of all mutants have a reduced virulence of log2(FC) −1.53 and log2(FC) of −2.75, respectively, and the other 97% are neutral in respect to virulence. The sensitivity reaches 99% at 40 detected mutants (lost virulence) and 100 detected mutants (reduced virulence), respectively. FC, fold change; iPool-Seq, insertion Pool-Sequence. (TIF) [file pbio.2005129.s005.tif]
