# Supplementary material for: In vivo insertion pool sequencing identifies virulence factors in a complex fungal–host interaction
Source: PLoS Biol. 2018 Apr 23;16(4):e2005129. doi: 10.1371/journal.pbio.2005129 (PMC5912717; doi:10.1371/journal.pbio.2005129)
Supplement: S1 Supporting methods — iPool-Seq, insertion Pool-Sequencing. (PDF) [file pbio.2005129.s007.pdf]

S1 SUPPORTING METHODS:
IPOOL-SEQ ANALYSIS PIPELINE DESCRIPTION

1. Read validation & mapping

Demultiplexing. The 12 libraries (one input and one output library for each of three replicates in experiments A & B) were sequenced (paired-end, 75 bp reads from both fragment ends) on two Illumina MiSeq flowcells (one per experiment). The runs were demultiplexed using *deML* [1] (pre-release, commit 80a491), and separate BAM files for each library are available in the *european nucleotide archive* (ENA), accession PRJEB23309.

Read-through removal. Read-throughs into the sequencing adapter on the other end (for short fragments) were removed using *Trimmomatic* [2] (version 0.33) in `PE` (paired-end) mode using commands `ILLUMINACLIP:adpaters.fa:2:24:15:1:true` and `MINLEN:40`, with `adapters.fa` containing the following two sequencing adapters:

```
>PrefixPE/1
CACGACGCTCTTCCGATCT
>PrefixPE/2
GTGACTGGAGTTCAGACGTGTGCTCTTCCGATCT
```

UMI extraction & technical sequence removal (`trim.tag.py`). From the construction of the 195 (single-gene) insertional mutants of *U. maydis* and the library preparation protocol used, we expected the double-stranded fragments subjected to sequencing to have the following layout (both strands shown):

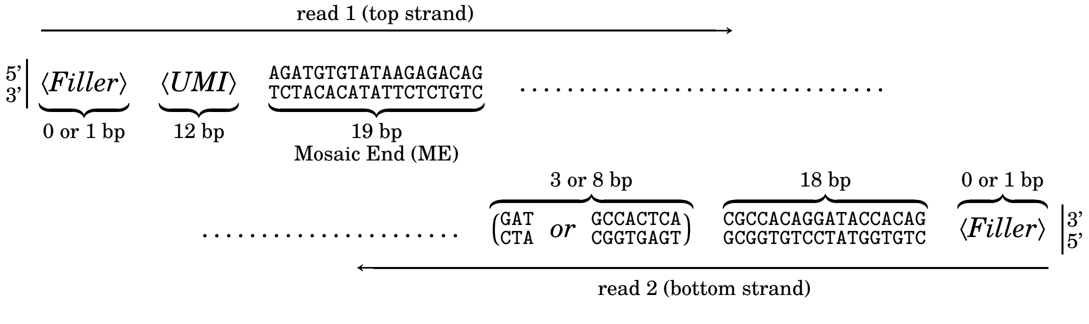

The part denoted "..." is a genomic *U. maydis* sequence, more specifically a sequence from the 3' or 5' flank of one the 195 studied genes. Our custom script `trim.tag.py` matched the sequenced read pairs against this expected pattern, allowing up to 4 mismatches (not counting `N`s) within the fixed part of each mate. Our script then stored the UMIs as part of the read names, and stripped all technical sequences (i.e. everything except the "..." part) from the reads. If the two mates of a pair overlapped (i.e. for fragments shorter than $2 \cdot 75 = 150$ bp), a technical sequence from one mate possibly appeared reverse-completed on the other mate as well. We detected this by checking whether a gap-less ends-free alignment of the two reads had an identity $\geq 90\%$, and then used the alignment to locate and remove the corresponding part of the complementary mate as well.

Assignment to mutants (`assign_to_features.py`)**.** To assign the reads to genes (and hence to insertional mutants), we mapped the paired-end reads (after UMI extraction and technical sequence removal) to the *U. maydis* genome, *GeneBank* accession GCF_000328475.2 [3], using *NextGenMap* [4] (version v0.4.13) with parameters `--end-to-end --pair-score-cutoff 0.5 --sensitivity 0.3 --kmer 13 --kmer-skip 0`.

Proper read pairs (read pairs where one mate maps in the forward direction, the other in the reverse direction, and the mates point "towards" one another) were assigned to a particular gene if either mate's first sequenced base mapped to within ±10 bp of one of the genes flanks, and the rest of that read continued "away" from the gene.

Improper read pairs (non-proper read pairs where nevertheless both mates were mapped) were ignored.

Singleton reads (i.e. reads whose mate could not be mapped) were assigned to a particular gene if their first sequenced base mapped to within a 1000 bp window on either side of the gene and they continued "towards" the gene.

Read pairs assigned to no or multiple genes were ignored.

2. UMI analysis & abundance estimation

Correcting UMIs for sequencing errors (`umicounts.tag.py`)**.** To count *U. maydis* insertional mutant genomes (i.e. cells), we counted the number of (sufficiently distinct, to protect against sequencing errors) combinations of UMI and mapping position within the reads mapping to a particular flank (3' or 5') of a particular gene. For the sake of brevity, *UMI* in the following denotes a *combination* of a particular 12 bp molecular barcode (so far called UMI) and the two mate's mapping positions.

To merge similar UMIs (which likely stem from the same cell), we used a variation of the algorithm of Smith *et al.* [5]. We started with the raw list of unique UMIs. We then marked an UMI p as *mergeable* into UMI q if the molecular barcodes disagreed at most at a single position, the mapping positions by no more than ±3 bases, and p was found in fewer reads than q. The UMIs not marked as mergeable were then assumed to be error-free. The read counts of UMIs that were mergeable (directly or indirectly) with a single error-free UMI were added to the error-free UMI's read count. UMIs marked (directly or indirectly) as mergeable with multiple error-free candidates were discarded as being ambiguous.

This produced, for both flanks of every gene, a separate list of assumedly error-free UMIs and per-UMI read counts.

Correcting for artifacts and lost UMIs to estimate abundance (`counts2results.R`)**.** We then further processed the per-flank UMIs using the algorithm of Pflug & von Haeseler [6], i.e. we removed all UMIs with a read count below a manually set read-count threshold ($T = 1$, except $T = 5$ for Experiment B R1 & R2 Output, and $T = 9$ for Experiment B R3 Output), and then estimated (for both flanks of every gene separately) the percentage ℓ of UMIs lost during sequencing and data filtering.

This yielded, separately for both flanks of every gene, a number n^{obs} of observed UMIs (after all filtering steps) and a loss estimate ℓ. Given these two, a (flank-specific) estimate of true mutant abundance is $n^{\text{obs}}/(1-\ell)$.

3. Statistical Analysis

Modelling growth of neutral mutants (`model.R`)**.** Given an insertional mutant m's *true* (unknown) abundances A_m^{in} and A_m^{out} in a particular pair of input and output libraries, and given the respective losses (i.e. fraction of unobserved or filtered UMIs) ℓ_{mf}^{in} and ℓ_{mf}^{out} for flank f (3' or 5'), we assumed that the observed

number of per-flank UMIs (after filtering) is Poisson distributed with mean $A_m^{\text{in}} \cdot (1-\ell_{mf}^{\text{in}})$ respectively $A_m^{\text{out}} \cdot (1-\ell_{mf}^{\text{out}})$. For the *sum* N_m^{in} respectively N_m^{out} of UMIs on the two flanks (5' and 3') of the mutamt m in the input respectively output library, it follows that

$$(1) \quad N_m^{\text{in}} \mid A_m^{\text{in}} \sim \text{Poisson}\left(A_m^{\text{in}} \cdot (1-\bar{\ell}_m^{\text{in}})\right), \quad N_m^{\text{out}} \mid A_m^{\text{out}} \sim \text{Poisson}\left(A_m^{\text{out}} \cdot (1-\bar{\ell}_m^{\text{out}})\right)$$
$$\text{where} \quad \bar{\ell}_m^{\text{in}} = \ell_{m,5'}^{\text{in}} + \ell_{m,3'}^{\text{in}}, \qquad \bar{\ell}_m^{\text{out}} = \ell_{m,5'}^{\text{out}} + \ell_{m,3'}^{\text{out}}.$$

We then further assumed that for neutral mutants the *expected* true input and output abundances are proportional (with the same factor λ for all neutral mutants in a particular pair of input and output libraries), but that the output abundances have additional dispersion d due to random fluctuations of mutant growth, i.e. that

$$(2) \qquad \mathbb{E}A_m^{\text{out}} = \lambda \cdot \mathbb{E}A_m^{\text{in}}, \qquad \mathbb{V}A_m^{\text{out}} = \lambda^2 \cdot \mathbb{V}A_m^{\text{in}} + d \cdot \left(\mathbb{E}A_m^{\text{out}}\right)^2.$$

To find the *null distribution* (i.e. assuming mutant m is neutral) for the output UMI count N_m^{out} given observed input count n_m^{in}, we computed the posterior $A_m^{\text{in}} \mid N_m^{\text{in}}$ (using degenerate prior Gamma(0,0)), added dispersion d to get $A_m^{\text{out}} \mid N_m^{\text{in}}$, and combined with $N_m^{\text{out}} \mid A_m^{\text{out}}$. The resulting *negative binomial* distribution depends on two mutant-independent parameters, proportionality factor λ and dispersion d,

$$(3) \qquad N_m^{\text{out}} \mid n_m^{\text{in}} \sim \text{NegBin}\left(\mu_m := \lambda \cdot n_m^{\text{in}} \cdot \frac{1-\bar{\ell}_m^{\text{out}}}{1-\bar{\ell}_m^{\text{in}}},\, r_m := \frac{n_m^{\text{in}}}{1+d \cdot n_m^{\text{in}}}\right).$$

Computing p-values, q-values and effect sizes (`r4896.Rmd`, `r5157.Rmd`). For each of the 6 pairs of input and output libraries, we estimated λ and d by maximizing the likelihood of the negative binomial model (3) over a reference set of neutral mutants (see below for how those were selected). Given λ and d, we then computed (one-sided) p-values p_m^{low} (sig. of depletion in output) and p_m^{high} (sig. enrichment in output), for each mutant m detected in both output and input, as

$$(4) \qquad p_m^{\text{low}} = \mathbb{P}\left(N_m^{\text{out}} \leq n_m^{\text{out}}\right), \quad p_m^{\text{high}} = \mathbb{P}\left(N_m^{\text{out}} \geq n_m^{\text{out}}\right) \quad \text{if } n_m^{\text{in}}, n_m^{\text{out}} \geq 1.$$

To control the *false discovery rate* (FDR), we applied the Benjamini-Hochberg (BH) procedure [7] (separately) to the collection of low and high p-values computed for a particular pair of input and output libraries, and set the FDR target to 10%.

To quantify the effect size, we also computed the $\log_2$ fold change (lfc_m) between each mutant m's observed output UMI count and the expected value for neutral mutants,

$$(5) \qquad \text{lfc}_m = \log_2 \frac{n_m^{\text{out}} \cdot (1-\bar{\ell}_m^{\text{in}})}{\lambda \cdot n_m^{\text{in}} \cdot (1-\bar{\ell}_m^{\text{out}})}.$$

Selecting the neutral reference set. We started with a candidate list of 13 insertional mutants described as neutral in the literature (UMAG_01297, UMAG_01300, UMAG_01302, UMAG_02192, UMAG_02193, UMAG_03046, UMAG_03201, UMAG_03202, UMAG_03615, UMAG_06222, UMAG_10403, UMAG_10553, UMAG_12313), estimated λ and d for all 6 input-output pairs, and computed these mutants' $\log_2$ fold changes. Suspecting that not all of these mutants are truly neutral, we looked for outliers (defined as for boxplots in R, values more than 1.5 IQR larger/smaller than the 75%/25% quantile) amongst these $\log_2$ fold changes and discarded them. We repeated this procedure for the remaining 8 candidates (UMAG_01302, UMAG_02192, UMAG_02193, UMAG_03046, UMAG_03202, UMAG_03615, UMAG_10403, UMAG_10553), and found 3 additional outliers. The remaining 5 candidate mutants (UMAG_01302, UMAG_02193, UMAG_03202, UMAG_10403, UMAG_10553) were then used as the final neutral reference set, and all p-values, q-values and $\log_2$ fold changes were re-computed based on this set.

Sensitivity of a genome-wide screen. To estimate the sensitivity of a genome-wide screen, we simulated experiments containing $m = 20,000$ distinct mutants using the statistical model from equation (1), but assuming a negative binomial distribution for N_m^{out} to account for the additional dispersion d of the output abundances (see also equation 2). We assumed the input abundances to be identical for all mutants (i.e $A_1^{\text{in}} = \ldots = A_{20.000}^{\text{in}} = A^{\text{in}}$), the output abundances of k mutants to show a virulence phenotype and hence to be reduced $2^{-\rho}$-fold (i.e. $A_1^{\text{out}} = \ldots = A_k^{\text{out}} = A^{\text{in}} \cdot 2^{\rho}$), and the other $m-k$ mutants to be neutral ($A_{k+1}^{\text{out}} = \ldots = A_{20,000}^{\text{out}} = A^{\text{in}}$). Based on $\approx 14\%$ of mutants in our screen showing a reproducible phenotype, and supplemental table 5 of Lanver *et al.* [8] showing $\approx 22\%$ of genes to be upregulated during infection, we set $k = 20,000 \cdot 0.14 \cdot 0.22 = 600$ (i.e. $\approx 3\%$ of mutants have a virulence phenotype). We set the additional dispersion d to the highest value observed in our 6 experiments (0.0126), and simulated 100 experiments for each input abundance $A^{\text{in}} = 1, 2, \ldots, 100$, once with $\log_2$ fold change of $\rho = -1.53$ (corresponding to the "Reduced" group in figure 4a) and once with $\rho = -2.75$ (corresponding to the "Lost virulence" group). For each simulated experiment we computed q-values as described above (see *Computing p-values, q-values and effect sizes*), determined the percentage of significant mutants within the ones with a virulence phenotype, and averaged these percentages over the 100 experiments to compute the efficiencies shown in figure S3.

4. Running the pipeline

Required software in addition to cited. *GNU Bash* (4.2.53). *GNU Make* (4.0). *Picard* (1.141). *samtools* (1.3.1). *gzip* (1.6). *python* (2.7.5). Python libraries: *recordtype* (1.1), *distance* (0.1.3), *regex* (2016.4.15), *pysam* (0.12.0.1), *bcbio-gff* (0.6.2), *biopython* (1.66). *R* (3.2.1). R libraries: *data.table* (1.10.4), *parallel* (3.2.1), *rmarkdown* (1.8). R Bioconductor Libraries: *rtracklayer* (1.30.4). Other R libraries: *gwpcR*[a] (0.9.9).

Running "abundance estimation" (incl. prerequisite steps). The pipeline (see S1 Software *iPool-Seq Analysis Pipeline*) uses separate subdirectories under `data/` for each library, e.g. `data/r4896.in1` for the input library of replicate 1 of experiment A. These directories contains various file controlling the pipeline (`tom.cfg`, `ngm.cfg`, `ref.fasta`, `features.gff`, `ngm.results.cfg`). To repeat our analyses, download the BAM files belonging to 12 libraries from `ftp://ftp.sra.ebi.ac.uk/vol1/ERA112/ERA1125781/bam/`, and store the file named r*<experiment_id>*/*<library>.bam* as `data/r`*<experiment_id>*`.`*<library>*`/raw.bam`.

The pipeline produces for each library two R data files as output, `ngm.results.rda` and `ngm.stats.rda`. For each subdirectory of `data/` run:

```
make data/<subdir>/ngm.results.rda data/<subdir>/ngm.stats.rda
```

Running "Statistical Analysis". The pipeline contains two R notebooks, `r4896.Rmd` (experiment A) and `r5157.Rmd` (experiment B). In R, run them with:

```
library(rmarkdown)
render("<experiment_id>.Rmd", output_format="pdf_document")
```

This produces a PDF report (`r`*<experiment_id>*`.pdf`) and table (`r`*<experiment_id>*`.abundance.csv`) listing for each mutant the raw and loss corrected input and output abundances, p- and q-values for significant depletion and enrichment, and the $\log_2$ fold change. It also produces two tables summarizing the significantly depleted (`r`*<experiment_id>*`.low.csv`) respectively enriched (`r`*<experiment_id>*`.high.csv`) mutants, and a R data file (`r`*<experiment_id>*`.model.rda`) containing the parameters of the null distributions.

[a] `http://github.com/Cibiv/gwpcR`, see also Pflug & von Haeseler [6]

References

1. Renaud G, Stenzel U, Maricic T, Wiebe V, Kelso J. deML: Robust demultiplexing of Illumina sequences using a likelihood-based approach. Bioinformatics. 2015;31(5):770–772. doi:10.1093/bioinformatics/btu719.
2. Bolger AM, Lohse M, Usadel B. Trimmomatic: A flexible trimmer for Illumina sequence data. Bioinformatics. 2014;30(15):2114–2120. doi:10.1093/bioinformatics/btu170.
3. Kämper J, Kahmann R, Bölker M, Ma LJ, Brefort T, Saville BJ, et al. Insights from the genome of the biotrophic fungal plant pathogen Ustilago maydis. Nature. 2006;444(7115):97–101. doi:10.1038/nature05248.
4. Sedlazeck FJ, Rescheneder P, von Haeseler A. NextGenMap: fast and accurate read mapping in highly polymorphic genomes. Bioinformatics. 2013;29(21):2790–2791. doi:10.1093/bioinformatics/btt468.
5. Smith T, Heger A, Sudbery I. UMI-tools: modeling sequencing errors in Unique Molecular Identifiers to improve quantification accuracy. Genome Res. 2017;27(3):491–499. doi:10.1101/gr.209601.116.
6. Pflug FG, von Haeseler A. TRUmiCount: Correctly counting absolute numbers of molecules using unique molecular identifiers; 2017. Preprint. Available from: `http://www.biorxiv.org/content/early/2017/11/13/217778`. Cited 13 November 2017. doi:10.1101/217778.
7. Benjamini Y, Hochberg Y. Controlling the false discovery rate: a practical and powerful approach to multiple testing. J R Stat Soc Series B Stat Methodol. 1995;57(1):289–300. doi:10.2307/2346101.
8. Lanver D, Müller AN, Happel P, Schweizer G, Haas FB, Franitza M, et al. The biotrophic development of Ustilago maydis studied by RNAseq analysis. Plant Cell. 2018;30(2):300–323. doi:10.1105/tpc.17.00764.
